# Dynamic Regulation of Myosin Light Chain Phosphorylation by Rho-kinase

**DOI:** 10.1371/journal.pone.0039269

**Published:** 2012-06-19

**Authors:** Takako Kaneko-Kawano, Fugo Takasu, Honda Naoki, Yuichi Sakumura, Shin Ishii, Takahiro Ueba, Akinori Eiyama, Aiko Okada, Yoji Kawano, Kenji Suzuki

**Affiliations:** 1 College of Pharmaceutical Sciences, Ritsumeikan University, Kusatsu, Shiga, Japan; 2 Department of Information and Computer Sciences, Nara Women’s University, Nara, Japan; 3 Department of Systems Science, Graduate School of Informatics, Kyoto University, Uji, Kyoto, Japan; 4 Department of Information Science and Technology, Aichi Prefectural University, Nagakute, Aichi, Japan; 5 Laboratory of Plant Molecular Genetics, Nara Institute of Science and Technology, Ikoma, Nara, Japan; Mayo Clinic, United States of America

## Abstract

Myosin light chain (MLC) phosphorylation plays important roles in various cellular functions such as cellular morphogenesis, motility, and smooth muscle contraction. MLC phosphorylation is determined by the balance between activities of Rho-associated kinase (Rho-kinase) and myosin phosphatase. An impaired balance between Rho-kinase and myosin phosphatase activities induces the abnormal sustained phosphorylation of MLC, which contributes to the pathogenesis of certain vascular diseases, such as vasospasm and hypertension. However, the dynamic principle of the system underlying the regulation of MLC phosphorylation remains to be clarified. Here, to elucidate this dynamic principle whereby Rho-kinase regulates MLC phosphorylation, we developed a mathematical model based on the behavior of thrombin-dependent MLC phosphorylation, which is regulated by the Rho-kinase signaling network. Through analyzing our mathematical model, we predict that MLC phosphorylation and myosin phosphatase activity exhibit bistability, and that a novel signaling pathway leading to the auto-activation of myosin phosphatase is required for the regulatory system of MLC phosphorylation. In addition, on the basis of experimental data, we propose that the auto-activation pathway of myosin phosphatase occurs *in vivo.* These results indicate that bistability of myosin phosphatase activity is responsible for the bistability of MLC phosphorylation, and the sustained phosphorylation of MLC is attributed to this feature of bistability.

## Introduction

Signaling networks regulate cellular functions through an interconnected set of proteins that transmit signaling information via the network. An impaired balance of signaling molecule activities induces several diseases. Accordingly, understanding the principle of system regulation of the balance of signaling molecules’ activities in cells is essential to establish new strategies for the prevention and treatment of diseases. However, it is difficult to monitor the balance of highly interconnected signaling molecule activities *in vivo*, and to elucidate the principles by which a system regulates the balance between activities of signaling molecules. Mathematical analysis has proven to be an efficient tool for the elucidation of these regulatory system mechanisms.

Small Rho GTPase and Rho-associated kinase (Rho-kinase) play an important role in the regulation of MLC phosphorylation [1]. Rho cycles between two conformational states, the GDP-bound (inactive) state and the GTP-bound (active) state [2]. Rho exerts its biological functions through interaction with specific effectors [3]. Rho-kinase is identified as an effector of Rho, and Rho activates Rho-kinase downstream of various extracellular signals [4]–[6]. There are two Rho-kinase members, Rho-kinase α/ROCK2/ROKα and Rho-kinase β/ROCK1/ROKβ: we will refer to them collectively as Rho-kinase [1]. Activated Rho-kinase directly phosphorylates MLC [7]. The phosphorylation of MLC is also regulated by myosin phosphatase, which consists of three subunits, a myosin phosphatase targeting subunit (MYPT1), a 20-kDa small subunit (M20), and a catalytic subunit of the type 1 protein serine/threonine phosphatase family, PP1δ[8]–[10]. MYPT1 is phosphorylated by Rho-kinase and thereby inactivates myosin phosphatase activity [11]. Therefore, Rho-kinase promotes actomyosin contraction in two ways: one by direct phosphorylation of MLC and the other by inactivation of myosin phosphatase through the phosphorylation of MYPT1 [1]. However, the system dynamics whereby Rho/Rho-kinase regulates MLC phosphorylation remains to be clarified.

Abnormal smooth muscle contraction causes diseases such as hypertension, vasospasm, and asthma [12]. The contractile state of smooth muscle is driven by phosphorylation of MLC. The sustained phosphorylation of MLC and MYPT1 are implicated in the pathogenesis of certain vascular diseases, including subarachnoid hemorrhage-induced cerebral vasospasm, coronary vasospasm, essential hypertension, and pulmonary hypertension [13], [14]. Rho-kinase dependent MLC phosphorylation is also involved in the regulation of endothelial permeability. When endothelial cells are stimulated with thrombin, MLC phosphorylation is sustained though Rho activation is transient [15]–[17]. Thrombin dependent sustained MLC phosphorylation is similar to prolonged phosphorylation of MLC at the lesion site in several vascular diseases. Understanding the system regulating the mechanism of MLC phosphorylation is fundamental to the prevention and treatment of diseases caused by abnormal smooth muscle contraction.

Dynamic analysis requires quantitative experimental data, for example, time dependent changes in activities and concentrations of signaling molecules *in vivo*. However, such quantitative experimental data have not been sufficient for the analysis of dynamics. In endothelial cells, Maeda and colleagues quantitatively measured thrombin-induced Rho activation and the phosphorylation of MLC and MYPT1 in human umbilical vein endothelial cells (HUVEC) [18]. A high concentration of thrombin (0.25 U/ml) induces sustained phosphorylation of MLC and MYPT1, whereas a low concentration of thrombin (0.05 U/ml) induces transient phosphorylation of MLC and MYPT1. On the other hand, both high and low thrombin stimuli induce transient Rho activation, which peaks at 1–2 minutes after thrombin stimuli, and maximal activity is about 10- and 4-fold over basal Rho activity, respectively. In each case, Rho activity quickly returns to basal levels after maximal activation [18]. In addition, Rho-kinase inhibitor (Y-27632) suppresses the sustained phosphorylation of MLC and MYPT1 induced by stimulation with a high concentration of thrombin [18]. To clarify the mechanism by which agonist induces the sustained phosphorylation of MLC, Maeda and colleagues performed computational simulation, incorporating signaling pathways already known to regulate MLC phosphorylation. However, this computational simulation did not reproduce thrombin-dependent sustained MLC phosphorylation [18]. These results suggest that an additional pathway(s) may be required for sustained MLC phosphorylation [18].

We reconsidered the signaling pathway regulating MLC phosphorylation through Rho/Rho-kinase. In common with MLC, other substrates of Rho-kinase (ERM family proteins, adducin, Tau, and MAP2) are regulated by Rho-kinase and myosin phosphatase [11], [19]–[21]. Since MYPT1 is also a substrate of Rho-kinase, it is possible that myosin phosphatase undergoes auto-dephosphorylation following phosphorylation by Rho-kinase. In fact, the phosphatase inhibitor Calyculin A, which suppresses the activity of protein phosphatase 1 (PP1) and 2A (PP2A), accelerates the phosphorylation of MYPT1 [22]–[24]. Kimura and colleagues have reported the direct dephosphorylation of MYPT1 by myosin phosphatase *in vitro* in the following experiment: in an *in vitro* phosphatase assay using purified myosin phosphatase, Rho-kinase, and MLC, they found that the purified myosin phosphatase holoenzyme directly dephosphorylates its regulatory subunit, MYPT1, which has been phosphorylated by Rho-kinase [25]. We speculate that myosin phosphatase auto-dephosphorylation may be the so-called additional pathway responsible for sustained MLC phosphorylation.

In the present study, to examine the characteristics of the system regulating MLC phosphorylation through Rho/Rho-kinase, we developed a mathematical model including a hypothetical pathway by which myosin phosphatase regulates MYPT1 phosphorylation by Rho-kinase. Our model reproduced the sustained phosphorylation of MLC and MYPT1 induced by transient Rho-kinase activation. Thus, myosin phosphatase auto-dephosphorylation, as an additional pathway, is considered to be a necessary component of the system regulating MLC phosphorylation by Rho/Rho-kinase. We also demonstrated experimentally that myosin phosphatase undergoes auto-dephosphorylation *in vivo.* Moreover, we showed that the activity of myosin phosphatase and MLC phosphorylation indicate bistability in our model. The bistable system can adopt either of two alternative steady states in response to small changes in stimulus [26]. Hence, we propose that bistability plays a critical role in the system, whereby Rho/Rho-kinase regulates MLC phosphorylation.

## Materials and Methods

### Mathematical Modeling, Parameters, and Simulation

**Figure 1:**
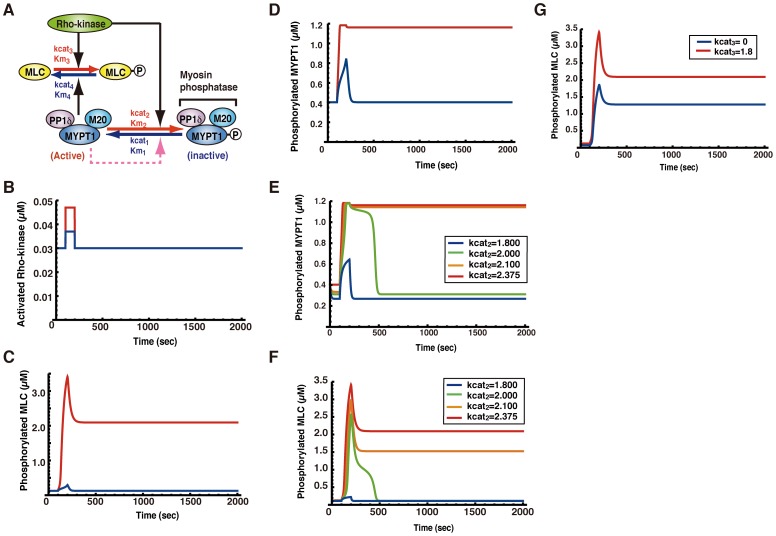
Mathematical modeling and simulation of MLC phosphorylation regulated by Rho-kinase. (A) Schematic overview of MLC phosphorylation regulated by Rho-kinase signal. Arrows, dashed line and - indicate stimulatory enzymatic reactions, myosin phosphatase auto-dephosphorylation signaling pathway and phosphorylation, respectively. (B) The graph shows that the transient Rho-kinase activations (red and blue line) are given as the input signal in our simulation. (C)(D) The simulation results of MLC and MYPT1 phosphorylation induced by the transient Rho-kinase activation in Figure 1B. (E)(F) The phosphorylation of MYPT1 and MLC against time t was calculated using the various kcat_2_ values. (G) The phosphorylation of MLC against time t was calculated using the different kcat_3_ values.

All calculations were performed using Mathematica 7 (Wolfram). All reactions are represented by enzymatic reactions [27]. The biochemical and enzymatic reactions used in the study are shown in Figure 1A. All parameters used in our model are listed with their values in Table I.

**Table 1:** Parameter values for mathematical model.

Parameter	Value	Comments
Km_1_	16 µM	Assumption
kcat_1_	3/sec	Assumption
Km_2_	0.1 µM	Feng *et al.* J Biol Chem. 274, 3744–3752, 1999
kcat_2_	2.357/sec	Feng *et al.* J Biol Chem. 274, 3744–3752, 1999
Km_3_	2.47 µM	Feng *et al.* J Biol Chem. 274, 3744–3752, 1999
kcat_3_	1.8/sec	Feng *et al.* J Biol Chem. 274, 3744–3752, 1999
Km_4_	16 µM	Ichikawa *et al.* J Biol Chem. 271, 4733–4740, 1996
kcat_4_	13.5/sec	Ichikawa *et al.* J Biol Chem. 271, 4733–4740, 1996
[RhoK]_total_	0.047 µM	Suzuki *et al.* Blood, 93(10), 3408–3417, 1999
[MLC]_total_	5 µM	Kuroda *et al.* In Foundations of System Biology 279–294, 2001
[MP]_total_	1.2 µM	Kuroda *et al.* In Foundations of System Biology 279–294, 2001, Maeda *et al.* Genes Cells 11, 1071–1083, 2006
k_1_	0.01 µM	Fit to experimental data
k_2_	0.01 µM	Fit to experimental data

### Myosin Phosphatase Activity

In our model, the activation of myosin phosphatase is described by the following combined equation composed of enzymatic reactions and a first order reaction:
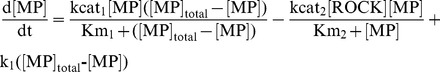
(1)where [] denotes the concentration of the molecules at time t. ROCK denotes the activated form of Rho-kinase. MP denotes activated myosin phosphatase, which contains non-phosphorylated MYPT1. The total concentration of myosin phosphatase ([MP]_total_) is conserved throughout the reaction. ([MP]_total_-[MP]) is the concentration of the inactive form of myosin phosphatase containing phosphorylated MYPT1. kcat_1_ and kcat_2_ are the turnover numbers of myosin phosphatase and Rho-kinase, respectively. Km_1_ and Km_2_ are Michaelis constants. k_1_ is the rate constant for the reaction. k_1_([MP]_total_-[MP]) assumes the activation of myosin phosphatase by other signaling pathways.

At steady state, the concentration of activated myosin phosphatase becomes constant. Therefore, by setting equation (1) equal to zero, we obtained equation (2).
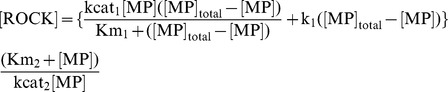
(2)


### Phosphorylation of MLC

Phosphorylation of MLC is also described by the equation consisting of the enzymatic reactions and the first order reaction:
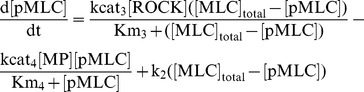
(3)where [] denotes the concentration of the molecules at time t. pMLC denotes phosphorylated MLC. The total concentration of MLC ([MLC]_total_) is conserved throughout the reaction. ([MLC]_total_-[pMLC]) denotes the concentration of non-phosphorylated MLC. kcat_3_ and kcat_4_ are the turnover numbers of Rho-kinase and myosin phosphatase, respectively. Km_3_ and Km_4_ are Michaelis constants of Rho-kinase and myosin phosphatase. k_2_ denotes the rate constant for the reaction. k_2_([MLC]_total_-[pMLC]) assumes the phosphorylation of MLC by other signaling pathways.

### Materials and Chemicals

Inhibitor and antibodies were obtained from commercial sources. Calyculin A was obtained from Wako Co. (Osaka, Japan). cDNA-encoding human CPI-17 was purchased from Invitrogen Co. (Carlsbad, CA, USA). pCAGGS-myc-kk1 vector was provided by Dr. K. Kaibuchi (Nagoya University, Nagoya, Japan).

### Plasmid Constructs

cDNA fragment encoding CPI-17 full length was amplified by polymerase chain reaction (PCR) using the obtained cloned as a template, containing the entire open reading frame of human CPI-17. The CPI-17 mutants, T38D and T38A, were generated with a site-directed mutagenesis kit (Stratagene, La Jolla, CA, USA) by replacing Thr-38 with Asp-38 or Ala-38. The cDNA fragments were subcloned into pCAGGS-myc-kk1 vector.

### Culture Preparation

HEK293 cells were grown in Dulbecco’s modified eagle medium (DMEM) containing 10% fetal bovine serum (FBS) at 37°C in an air-5% CO_2_ atmosphere at constant humidity. Transfection of plasmid into cells was carried out using Hillymax reagent (Dojindo, Kumamoto, Japan) according to the manufacturer’s protocol. For the treatment of Calyculin A, cells were seeded onto 6 well plates coated with poly-D-lysine (PDL) and cultured overnight.

### MYPT1 Phosphorylation

After treatment with 0.1 µM Calyculin A for 10 min and transfection, cells were harvested with 10% (w/v) trichloroacetic acid. The resulting precipitates were subjected to immunoblotting with anti-phospho-MYPT1 (Thr-853) antibody (Millipore, Billerica, MA, USA), anti-MYPT1 antibody (BD, Lakes, NJ, USA), and anti-myc antibody (Millipore, Billerica, MA, USA).

## Results

### Modeling the System Responsible for Regulation of MLC Phosphorylation by Rho/Rho-kinase Signaling

To examine the dynamics whereby Rho/Rho-kinase regulates MLC phosphorylation, we constructed the mathematical model based on previous experimental results showing thrombin-dependent MLC phosphorylation in endothelial cells [18]. Our simple mathematical model incorporates only three components, Rho-kinase, myosin phosphatase, and MLC **(** **Figure 1A** **)**. Rho-kinase phosphorylates MYPT1, a regulatory subunit of myosin phosphatase. The activity of myosin phosphatase is suppressed through MYPT1 phosphorylation by Rho-kinase. Myosin phosphatase incorporating non-phosphorylated MYPT1 can be regarded as an active form of myosin phosphatase. In our model, the concentration of non-phosphorylated MYPT1 is considered to represent that of activated myosin phosphatase. Some substrates of Rho-kinase are regulated by myosin phosphatase, and MYPT1 is a substrate of Rho-kinase. Thus, we assumed that myosin phosphatase dephosphorylates MYPT1, as a hypothetical pathway (myosin phosphatase auto-dephosphorylation pathway) **(Dashed line in** **Figure 1A** **)**. Mathematical details of the model are provided in the Materials and Methods and Table I, along with a list of parameters and the basis on which they were chosen. We determined the kinetic parameters based on experimental observations and some assumptions **(Table I)**.

### A Myosin Phosphatase Auto-dephosphorylation Pathway is Required for the Sustained Phosphorylation of MLC

To test the validity of our mathematical model, the simulation results were compared with the already-known experimental data (phosphorylation profiles of MLC and MYPT1). It has been reported that stimulation with a high concentration of thrombin gives rise to sustained phosphorylation of MLC and MYPT1 *in vivo*, whereas stimulation with a low concentration leads only to transient phosphorylation of MLC and MYPT1 [18]. In contrast, stimulation with 0.25 U/ml (high) or 0.05 U/ml (low) thrombin yields high or low peaks of transient Rho activation *in vivo* [18]. Therefore, for simplification of our model, the high and low peak transient activities of Rho-kinase were applied to our simulation as the input signals. For each signal, 0.030 µM activated Rho-kinase was used in our simulation as the set of initial conditions **(** **Figure 1B** **)**. With the high peak activation signal, the concentration of activated Rho-kinase was transiently raised from 0.030 µM to 0.047 µM for only 100 seconds (100–200 sec) **(Red line in** **Figure 1B** **)**. On the other hand, with the low peak activation signal, the concentration of activated Rho-kinase was increased from 0.030 µM to 0.037 µM over the same 100 second interval (100–200 sec) **(Blue line in** **Figure 1B** **)**. Each signal returned to the basal level of Rho-kinase activity (0.030 µM) after transient activation.

We analyzed the dynamic behavior of MLC phosphorylation. We simulated the time course of phosphorylation of MLC according to equations (1) and (3) in Materials and Methods. The transient Rho-kinase activations are described in Figure 1B. When the transient Rho-kinase activations were used, the phosphorylation of MLC against time t was simulated using equations (1) and (3) **(** **Figure 1C** **)**. We used 0.1 µM phosphorylated MLC as the initial condition. The low peak activation of Rho-kinase transiently increased phosphorylation of MLC, which then returned to the basal level of phosphorylation **(Blue line in** **Figure 1C** **)**. In contrast, the high peak activation of Rho-kinase also increased the phosphorylation of MLC, which was maintained **(Red line in** **Figure 1C** **)**. Correspondingly, we analyzed the dynamic behavior of MYPT1 phosphorylation. With high and low transient activation of Rho-kinase, the phosphorylation of MYPT1 against time t was simulated using equation (1) **(** **Figure 1D** **)**. We used 0.86 µM activated myosin phosphatase as an initial condition. The high peak activation of Rho-kinase induced the sustained phosphorylation of MYPT1, whereas low peak activation of Rho-kinase transiently increased the phosphorylation of MYPT1 **(Blue and Red lines in** **Figure 1D** **)**. These results are consistent with the experimental dynamics of thrombin-induced phosphorylation of MLC and MYPT1 [18].

### Sustained Phosphorylation of MLC and MYPT1 is Suppressed by Inhibition of Rho-kinase Activity

It has been reported that the sustained phosphorylation of MLC and that of MYPT1 are repressed by the Rho-kinase specific inhibitor Y-27632 *in vivo* [18]. To examine the effects of Rho-kinase inhibition, we performed stepwise changes in the turnover number (kcat_2_ in Figure 1A) value of Rho-kinase towards MYPT1 in our model. The phosphorylation of MYPT1 against time t was calculated using the various kcat_2_ values (kcat_2_ = 1.800, 2.000, 2.100, and 2.375) of Rho-kinase, using equation (1) in Materials and Methods **(** **Figure 1E** **)**. Correspondingly, the phosphorylation of MLC against time t was calculated using the various kcat_2_ values (kcat_2_ = 1.800, 2.000, 2.100, and 2.375) for phosphorylation of MYPT1 by Rho-kinase, using equations (1) and (3) in Materials and Methods **(** **Figure 1F** **)**. In these simulations, the transient high peak of Rho-kinase activity was used **(Red line in** **Figure 1B** **)**. We showed that the phosphorylation of MYPT1 and MLC was altered from sustained to transient by the stepwise reduction in Rho-kinase turnover number (kcat_2_) towards MYPT1 **(** **Figure 1E and F** **)**. Furthermore, we demonstrated that a slight increase in turnover number produced sustained phosphorylation of MYPT1 and MLC **(Orange and green lines in** **Figure 1E and F** **)**. These results suggest that there is a threshold, above which there is a sustained MLC phosphorylation response, in this reaction. We also assessed the effect of suppression of the turnover number (kcat_3_ in Figure 1A) value of Rho-kinase for direct phosphorylation of MLC in our simulation. Furthermore, the phosphorylation of MLC against time t was calculated using the various kcat_3_ values (kcat_3_ = 0 and 1.8) for direct phosphorylation of MLC by Rho-kinase, and using equations (1) and (3) in Materials and Methods **(** **Figure 1G** **)**. Despite the suppression of direct MLC phosphorylation by Rho-kinase, MLC phosphorylation was maintained, even though phosphorylation level in the sustained phase was low **(** **Figure 1G** **)**. As compared with the results of the suppression of kcat_2_ and kcat_3_ **(** **Figure 1F and G** **)**, the suppression of kcat_2_ has an influence on the sustained MLC phosphorylation. On the regulation of MLC phosphorylation by Rho-kinase, our simulation results suggest that the major effect of Rho-kinase is suppression of myosin phosphatase activity rather than direct MLC phosphorylation. These results may agree with the previous observation that myosin phosphatase plays a pivotal role in controlling phosphorylation of MLC in response to physiological stimuli. We reproduced the sustained MLC phosphorylation induced by transient activation of Rho-kinase; this is a signal upstream of MLC, in our simulation, incorporating the hypothesis that myosin phosphatase dephosphorylates its regulatory subunit, MYPT1. On the basis of our simulation results, it is reasonable to assume that MYPT1 is dephosphorylated by myosin phosphatase in our mathematical model.

### Myosin Phosphatase Regulates Phosphorylation of MYPT1 In Vivo

**Figure 2:**
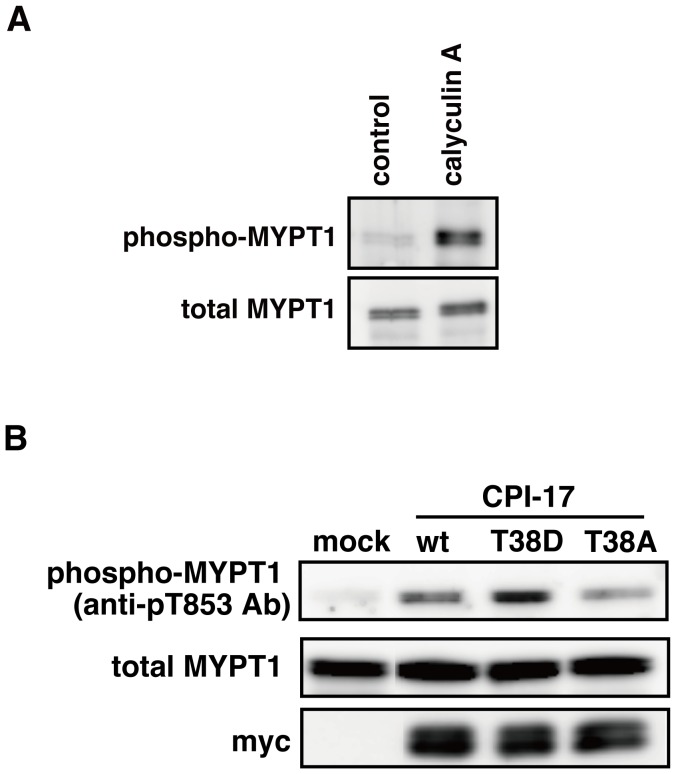
Inhibition of myosin phosphatase induces phosphorylation of MYPT1. (A) HEK293 cells were treated with 0.1 µM Calyculin A. Cell lysates were resolved by SDS-PAGE and immunoblotted with anti-phospho-MYPT1 (Thr-853), and anti-MYPT1 antibodies. (B) The lysates of cells expressing the indicated proteins were immunoblotted with anti-phospho-MYPT1 (Thr-853), anti-MYPT1, and anti-myc antibodies.

We determined experimentally whether MYPT1 is dephosphorylated by myosin phosphatase, using a phosphatase inhibitor and myosin phosphatase inhibitory protein. Calyculin A is an inhibitor of protein phosphatase 1 (PP1) and 2A (PP2A), and myosin phosphatase is a PP1. We found that Calyculin A induced an increase in MYPT1 phosphorylation at Thr-853, which is a phosphorylation site of Rho-kinase **(** **Figure 2A** **)**. These results indicate that MYPT1 is dephosphorylated by PP1 or PP2A, in agreement with previous reports [22]–[24]. It is known that a 17 kDa protein kinase C (PKC)-potentiated phosphatase inhibitory protein (CPI-17), phosphorylated at Thr-38, interacts with a catalytic subunit of myosin phosphatase and suppresses myosin phosphatase activity [28]. Thus, CPI-17 is considered to act as an inhibitory protein for myosin phosphatase. We produced CPI-17 mutants (CPI-17 T38D and T38A), in which Thr-38 was replaced by Asp or Ala. The expression of CPI-17 T38D, which was expected to mimic the behaviour of phosphorylated CPI-17, clearly induced an increase of MYPT1 phosphorylation at Thr-853, whereas CPI-17 WT and T38A only slightly increased the phosphorylation of MYPT1 **(** **Figure 2B** **)**. These results indicate that myosin phosphatase regulates the phosphorylation level of MYPT1. Therefore, it is appropriate to assume that myosin phosphatase directly dephosphorylates MYPT1 in our model.

### Bistable Response of Myosin Phosphatase Activity

**Figure 3:**
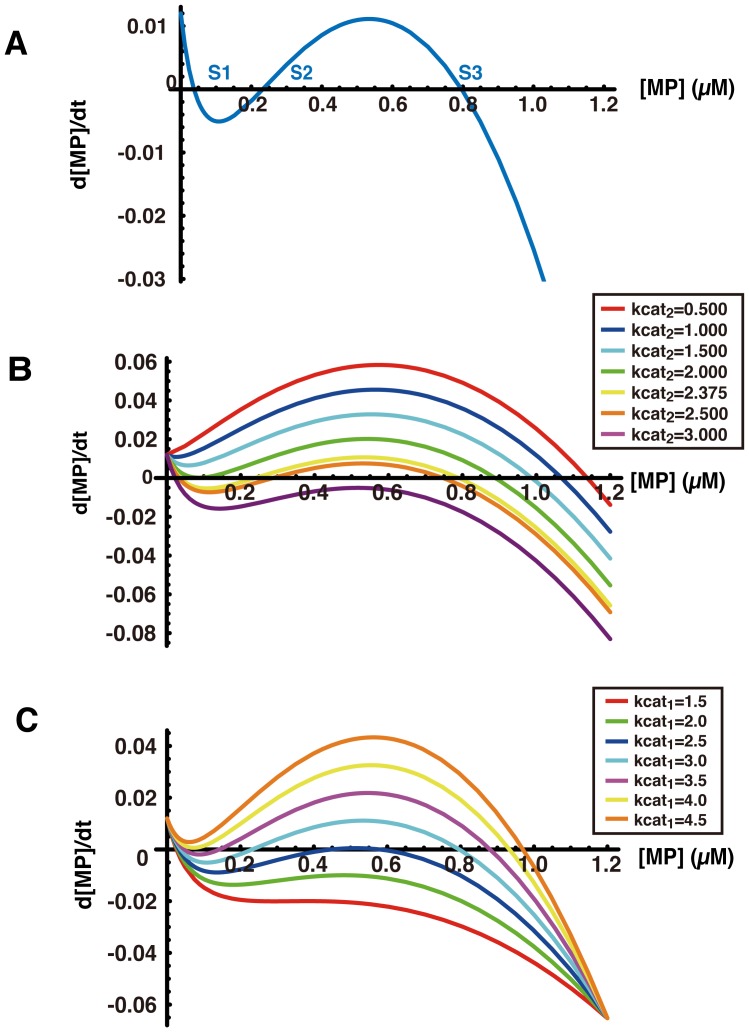
Activity of myosin phosphatase is bistable. [MP] denotes the concentration of activated myosin phosphatase. The concentration of activated myosin phosphatase is assumed to represent that of non-phosphorylated MYPT1. d[MP]/dt indicates the differentiation of [MP] against time t. (A) The plots are d[MP]/dt against [MP]. In the steady states, the reaction velocity is zero, whereby the steady states are d[MP]/dt = 0. Three intersections with the horizontal axis represent three steady states (S1, S2, and S3). (B) d[MP]/dt was plotted against [MP] using the various kcat_2_ values. (C) d[MP]/dt was plotted against [MP] using the various kcat_1_ values.

To investigate the dynamics whereby Rho/Rho-kinase regulates the phosphorylation of MLC, we analyzed the steady states of myosin phosphatase activity using the mathematical model. Since MYPT1 phosphorylated by Rho-kinase decreases its phosphatase activity, myosin phosphatase consisting of non-phosphorylated MYPT1 is assumed to represent the active form. We plotted the concentration of myosin phosphatase active form, denoted [MP], against its derivative with respect to time, which is denoted by d[MP]/dt using equation (1) in Materials and Methods, when activated Rho-kinase is 0.030 µM **(** **Figure 3A** **)**. At the steady states, the concentration of activated myosin phosphatase, [MP], is constant. In other words, the reaction velocity is zero, which means that the differentiation of activated myosin phosphatase concentration against time, d[MP]/dt, is zero. The plots showed the presence of three intersections with the horizontal axis **(S1, S2, and S3 in** **Figure 3A** **)**, each of which corresponds to a steady state, because the rate of change at these points is zero **(** **Figure 3A** **)**. d[MP]/dt exhibits the rate of change of [MP], for example, d[MP]/dt >0 indicates an increase in concentration of [MP], whereas d[MP]/dt <0 indicates a decline in [MP]. The plots indicate that [MP] converges to S1 or S3, when the system is given a slight perturbation in the vicinity of S1 and S3. However, the perturbation around S2 induces convergence to S1 or S3, but not S2. Thus, the system acquired two stable steady states, S1 and S3, separated by the unstable steady state S2 **(** **Figure 3A** **)**. These results suggest that the activity of myosin phosphatase is bistable in our model under these conditions.

A bistable system toggles between two discrete alternative stable steady states, in contrast to a monostable system, which slides along a continuum of steady states [29]–[36]. If myosin phosphatase activity is bistable, its activity converges to one of two stable states depending on initial state or external perturbations. Considering the behavior of a bistable system, if the activation of Rho/Rho-kinase exceeds a threshold due to stimulus by an extracellular signal, for example thrombin, it may trigger transition from the upper to the lower stable steady state of myosin phosphatase activity. Once the transition from the higher to the lower stable steady state occurs, the low activity of myosin phosphatase is maintained, even if the activation of Rho/Rho-kinase is decreased. It is possible that an irreversible transition of myosin phosphatase activity results in sustained MLC phosphorylation. The Rho-kinase specific inhibitor Y-27632 suppresses the sustained phosphorylation of MLC and MYPT1 *in vivo* [18]. To characterize the steady states of myosin phosphatase activity against Rho-kinase activity, d[MP]/dt was plotted against [MP] using the various kcat_2_ values (kcat_2_ = 0.500, 1.000, 1.500, 2.000, 2.375, 2.500 and 3.000) of Rho-kinase against MYPT1 **(** **Figure 3B** **)**. The number of intersections with the horizontal axis was altered from three to one by the stepwise change of kcat_2_ value **(** **Figure 3B** **)**. To characterize the steady states of myosin phosphatase activity against Rho-kinase, d[MP]/dt was plotted against [MP] using the various Michaelis constant values (Km_2_ = 0, 0.02, 0.04, 0.06, 0.08, 0.10, and 1.02) for phosphorylation of MYPT1 by Rho-kinase, and using equation (1) in Materials and Methods **(Figure S1A)**. The stepwise change of Km_2_ value also altered the number of intersections with the horizontal axis from three to one **(Figure S1A)**. These results suggest that Rho-kinase inhibition altered the system from bistable to monostable, thereby inhibiting sustained MLC phosphorylation.

We assumed that MYPT1 is dephosphorylated by myosin phosphatase, representing a myosin phosphatase auto-dephosphorylation signaling pathway. To identify the steady states of myosin phosphatase activity against the strength of the myosin phosphatase auto-dephosphorylation signal pathway, d[MP]/dt was plotted against [MP] using the various kcat_1_ values (kcat_1_ = 1.5, 2.0, 2.5, 3.0, 3.5, 4.0, and 4.5) of myosin phosphatase (for dephosphorylation of MYPT1), and using equation (1) in Materials and Methods **(** **Figure 3C** **)**. Likewise, to examine the steady states of myosin phosphatase activity against Rho-kinase activity, d[MP]/dt was plotted against [MP] using the various Km_1_ values (Km_1_ = 10, 12, 14, 16, 18, 20, and 22) for dephosphorylation of MYPT1 by myosin phosphatase, and also using equation (1) in Materials and Methods **(Figure S1B)**. The number of intersections with the horizontal axis was shifted from three to one by the stepwise change of kcat_1_ and Km_1_ value **(** **Figure 3C** **and Figure S1B)**. These results suggest that the system was changed from bistable to monostable in proportion to the extent of involvement of the myosin phosphatase auto-dephosphorylation signaling pathway.

### Bistability of Myosin Phosphatase Activity Induces Sustained MLC Phosphorylation

**Figure 4:**
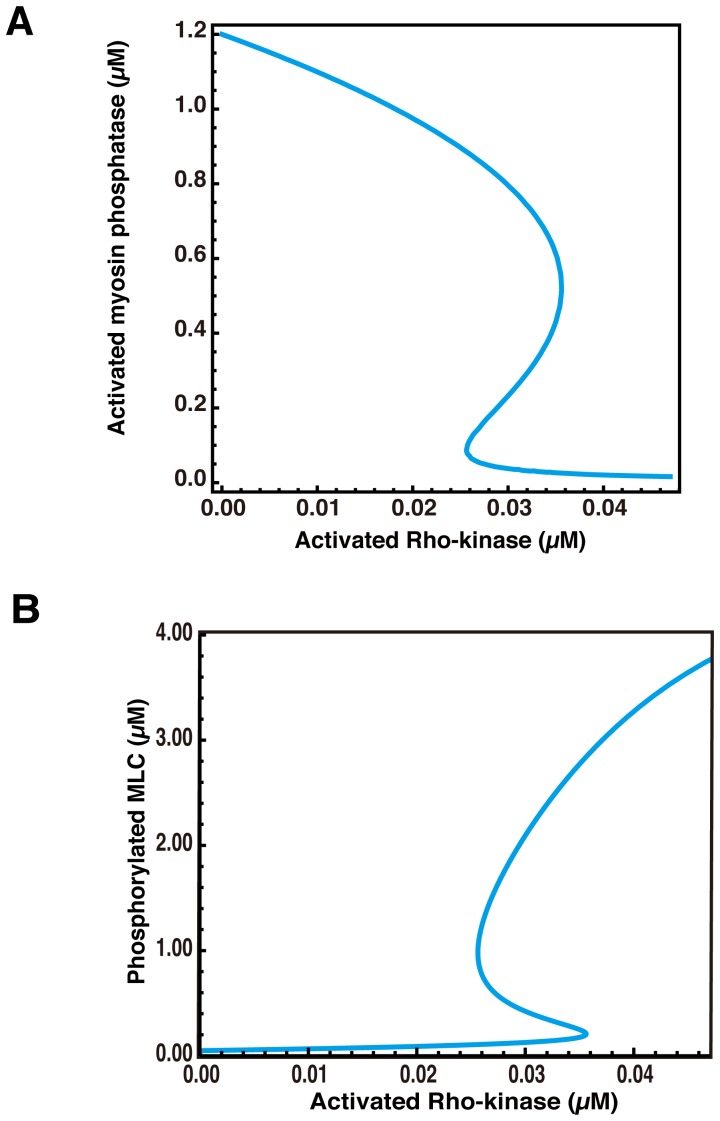
Bistability of myosin phosphatase activity induces sustained phosphorylation of MLC. (A) The bifurcation diagram shows the steady states of activated myosin phosphatase against Rho-kinase activity. The longitudinal axis is the steady state of myosin phosphatase activity. The horizontal axis shows the concentration of activated Rho-kinase. (B) The bifurcation diagram indicates the steady states of phosphorylated MLC against Rho-kinase activities. The longitudinal axis shows the steady state of phosphorylated MLC. The horizontal axis shows the concentration of activated Rho-kinase.

We first examined the relationship between Rho-kinase activity and the steady states of myosin phosphatase activity in our mathematical model. We obtained a bifurcation diagram from equation (2) in Materials and Methods **(** **Figure 4A** **)**. The bifurcation diagram revealed the steady states of activated myosin phosphatase ([MP]) against Rho-kinase activities ([ROCK]) **(** **Figure 4A** **)**. The hysteretic behavior of the system can be deduced from the results shown in **Figure 4A**; the elevation of Rho-kinase activity from low to high leads to the lower branch in the bistable curve of stable states of activated myosin phosphatase, whereas decreasing from high to low takes myosin phosphatase activity on the upper branch. These results suggest that myosin phosphatase activity is bistable. To evaluate the range of Rho-kinase activities leading to the bistability of myosin phosphatase activity, the steady state of myosin phosphatase activity was plotted against Rho-kinase activity and the rate constant (k_1_). This plot suggests that a wide range of Rho-kinase activities induce myosin phosphatase activity **(Figure S2)**.

Secondly, to investigate the correlation between the steady states of MLC phosphorylation and Rho-kinase activity, we obtained the value of [MP] against [ROCK] from equation (2) in Materials and Methods. At the steady state, the concentrations of phosphorylated MLC ([pMLC]) become constant. By setting equation (3) in Materials and Methods equal to zero and substituting the value of [ROCK] and [MP] obtained from equation (2) into equation (3), [pMLC] was calculated. The bifurcation diagram was obtained by plotting [pMLC] against [ROCK] **(** **Figure 4B** **)**. The bifurcation diagram indicated the bistability of MLC phosphorylation against Rho-kinase activity. Therefore, the bistability of myosin phosphatase activity leads to the bistable phosphorylation of MLC, and the property of bistability is responsible for the sustained phosphorylation. These results suggest that the steady state of MLC phosphorylation converges from low to high in excess of the threshold, due to the transient Rho-kinase activity. Consequently, transient Rho-kinase activation induces sustained MLC phosphorylation; the pharmacological action of a Rho-kinase inhibitor induces the transition from high to low steady state of MLC phosphorylation.

## Discussion

In this study, to elucidate the system dynamics, we developed a mathematical model in which Rho/Rho-kinase regulates MLC phosphorylation. We hypothesized that myosin phosphatase dephosphorylates MYPT1 phosphorylated by Rho-kinase. We demonstrated that our mathematical model incorporating the myosin phosphatase auto-dephosphorylation pathway reproduces the sustained phosphorylation of MLC and MYPT1 induced by transient activation of Rho-kinase. These results support our hypothesis that myosin phosphatase dephosphorylates the regulatory subunit of MYPT1. We showed that the activity of myosin phosphatase and MLC phosphorylation are bistable. Furthermore, the transition of myosin phosphatase activity from monostable to bistable depends on Rho-kinase. The bistability of myosin phosphatase activity is responsible for the bistability of MLC phosphorylation, and the sustained phosphorylation of MLC is attributed to the feature of bistability.

MLC phosphorylation status is determined by the balance between activities of kinases including Ca^2+^/calmodulin-dependent MLC kinase (MLCK) and Rho-kinase, and myosin phosphatase [37], [38]. Several agonists induce biphasic MLC phosphorylation, which consists of initial (phasic) and sustained (tonic) phases. The initial phosphorylation of MLC is regulated by Ca^2+^/Calmodulin-dependent MLCK signaling, whereas the sustained phosphorylation of MLC is regulated by Rho/Rho-kinase signaling [18], [39]–[41]. Previous studies have shown that MLCK inhibitor ML-9, selectively suppresses transient initial MLC phosphorylation, but not sustained MLC phosphorylation [41]. In contrast, Rho-kinase inhibitor significantly suppresses the sustained phosphorylation of MLC [18]. These results indicate that MLCK does not contribute to sustained MLC phosphorylation, and that Rho-kinase is a major regulator of MLC sustained phosphorylation. Since we focused on sustained MLC phosphorylation, our mathematical model does not include MLCK signaling. It is known that CPI-17 inhibits myosin phosphatase in a PKC-mediated phosphorylation-dependent manner [42]. However, thrombin does not induce significant phosphorylation of CPI-17 in endothelial cells [43]. Our model was based on the experimental results of thrombin-dependent MLC phosphorylation in endothelial cells. Thus, the CPI-17 signaling pathway was excluded from our mathematical model.

Rho-kinase has been reported to phosphorylate MYPT1 at Thr-696 and Thr-853 [1]. The phosphorylation of Thr-696 and Thr-853 in MYPT1 inhibits myosin phosphatase activity and interaction with myosin [44]–[47]. In smooth muscle and non-muscle cells, myosin phosphatase catalyses the reactions of dephosphorylation of MLC at Ser-19, regulating actomyosin relaxation [12]. The sequence of the regulatory phosphorylation site in MYPT1 around Thr-696 and Thr-853 resembles that of MLC around Ser-19 [48]. Therefore, this sequence is also a good candidate for binding at the catalytic cleft of myosin phosphatase, since charge complementarity exists between this sequence and the catalytic cleft of myosin phosphatase [48]. It is likely that myosin phosphatase controls phosphorylation of MYPT1 at Thr-696 or Thr-853. Furthermore, Calyculin A, an inhibitor of PP1 and PP2A, increases the phosphorylation of MYPT1 at Thr-696 and Thr-853 *in vivo* [22]–[24]. We confirmed that treatment with Calyculin A induced the phosphorylation of MYPT1 at Thr-853 **(** **Figure 2A** **)**. These results suggest that the phosphorylation of MYPT1 is regulated by PP1 or PP2A. Since myosin phosphatase is a PP1, these previous observations support the hypothesis that myosin phosphatase regulates the phosphorylation of MYPT1. Moreover, we found that the phosphorylation of MYPT1 was elevated by overexpression of CPI-17 phospho-mimic mutant (CPI-17 T38D), which suppresses myosin phosphatase activity **(** **Figure 2B** **)**. In addition to our experimental data, Kimura and colleagues have reported that the purified holoenzyme of myosin phosphatase exhibits phosphatase activity toward myosin phosphatase phosphorylated by Rho-kinase *in vitro* [25]. This result strongly indicates that myosin phosphatase directly dephosphorylates itself. Taken together, it is appropriate to assume that myosin phosphatase dephosphorylates MYPT1 in our model, resulting in reproduction of the sustained MLC phosphorylation induced by transient Rho-kinase activation.

A bistable signaling system must include a positive feedback loop, a double-negative feedback loop, or the equivalent [26]. However, the presence of positive or negative feedback does not guarantee that a system will be bistable. The positive and negative feedback loops exhibit bistability in parameter dependent manners. In our model, the myosin phosphatase auto-dephosphorylation signaling pathway serves as a positive feedback loop, because Rho-kinase suppresses myosin phosphatase activity through phosphorylation of MYPT1. We used the parameters obtained from the previous experimental observations as far as possible **(Table I)**, whereby the activity of myosin phosphatase indicates bistability. Since the phosphorylation of MLC is regulated by myosin phosphatase, the phosphorylation of MLC also exhibits bistability. ERM family proteins, adducin, Tau and MAP2 are found to be substrates of both Rho-kinase and myosin phosphatase [11], [19]–[21]. It is possible that the phosphorylation of these substrates also exhibits bistability. However, further study is necessary to address whether phosphorylation of these substrates may exhibit bistability.

Bistability can adopt either of two alternative steady states in response to a constant stimulus, and can toggle between alternative stable steady states in response to small changes in stimulus or transient stimulus [26]. A bistable system will convert a transient trigger stimulus into an irreversible response. In *Xenopus* oocyte maturation, ERK1/2 achieves a bistable form following a single stimulation with progesterone [49]. The bistability of MLC phosphorylation toggles between alternative stable steady states in response to small changes in stimulus and transient supra-threshold Rho/Rho-kinase activity. This prediction agrees with the experimental observation that transient Rho activation induced sustained phosphorylation of MLC. The bistable system is resistant to perturbations; it may robustly regulate the phosphorylation of MLC and the activity of myosin phosphatase.

Rho-kinase specific inhibitor Y-27632 suppresses the sustained phosphorylation of MLC and MYPT1 *in vivo* [18]. Rho-kinase inhibitors appear to be useful for treating disorders of vascular smooth muscle cells (e.g. hypercontraction, and arteriosclerotic diseases), and other smooth muscle cell types (e.g. bronchial asthma and glaucoma) [50]. It is possible that MLC phosphorylation usually converges to a lower stable steady state, but to a higher steady state at the lesion site. Some perturbation may cause the transition from a lower to a higher steady state at the lesion site. When Rho-kinase activity is gradually decreased, MLC phosphorylation abruptly transits from a higher to a lower steady state at the threshold **(** **Figure 4B** **)**. Therefore, the pharmacological action of Rho-kinase specific inhibitors may cause transition from a higher to a lower steady state.

Understanding the dynamics regulating MLC phosphorylation is essential to establish new strategies for the prevention and treatment of diseases of blood vessels and several other areas. This study sheds light on the dynamics underlying the regulation of MLC phosphorylation by Rho/Rho-kinase signaling. We predict that the positive feedback loop is required for the sustained MLC phosphorylation, and that the sustained MLC phosphorylation is regulated by bistability of myosin phosphatase activity. However, further experimental studies are required to determine whether myosin phosphatase dephosphorylates MYPT1 phosphorylated by Rho-kinase, and whether the phosphorylation of MLC and MYPT1 indicates bistability *in vivo.* Understanding the dynamics by which Rho/Rho-kinase regulates MLC phosphorylation in living cells will be a challenge for further experimental investigations.

## Supporting Information

Figure S1 **Effects of Rho-kinase and hypothetical pathway.** (A) d[MP]/dt was plotted against [MP] using the various Km_2_ values (Km_2_ = 0, 0.02, 0.04, 0.06, 0.08, 0.10, and 1.02) for phosphorylation of MYPT1 by Rho-kinase. (B) d[MP]/dt was plotted against [MP] using the various Km_1_ values (Km_1_ = 10, 12, 14, 16, 18, 20 and 22) for dephosphorylation of MYPT1 by myosin phosphatase.
(TIF)

Figure S2 **Estimation of Rho-kinase activity and rate constant k_1._** The steady states of activated myosin phosphatase against Rho-kinase activity and rate constant (k_1_). X, Y, and Z axes show the Rho-kinase activities, rate constant k_1_, and the steady states of myosin phosphatase, respectively.
(TIF)
