# Supplementary figures and images for: Dynamic Regulation of Myosin Light Chain Phosphorylation by Rho-kinase
Source: PLoS One. 2012 Jun 19;7(6):e39269. doi: 10.1371/journal.pone.0039269 (PMC3378528; doi:10.1371/journal.pone.0039269)

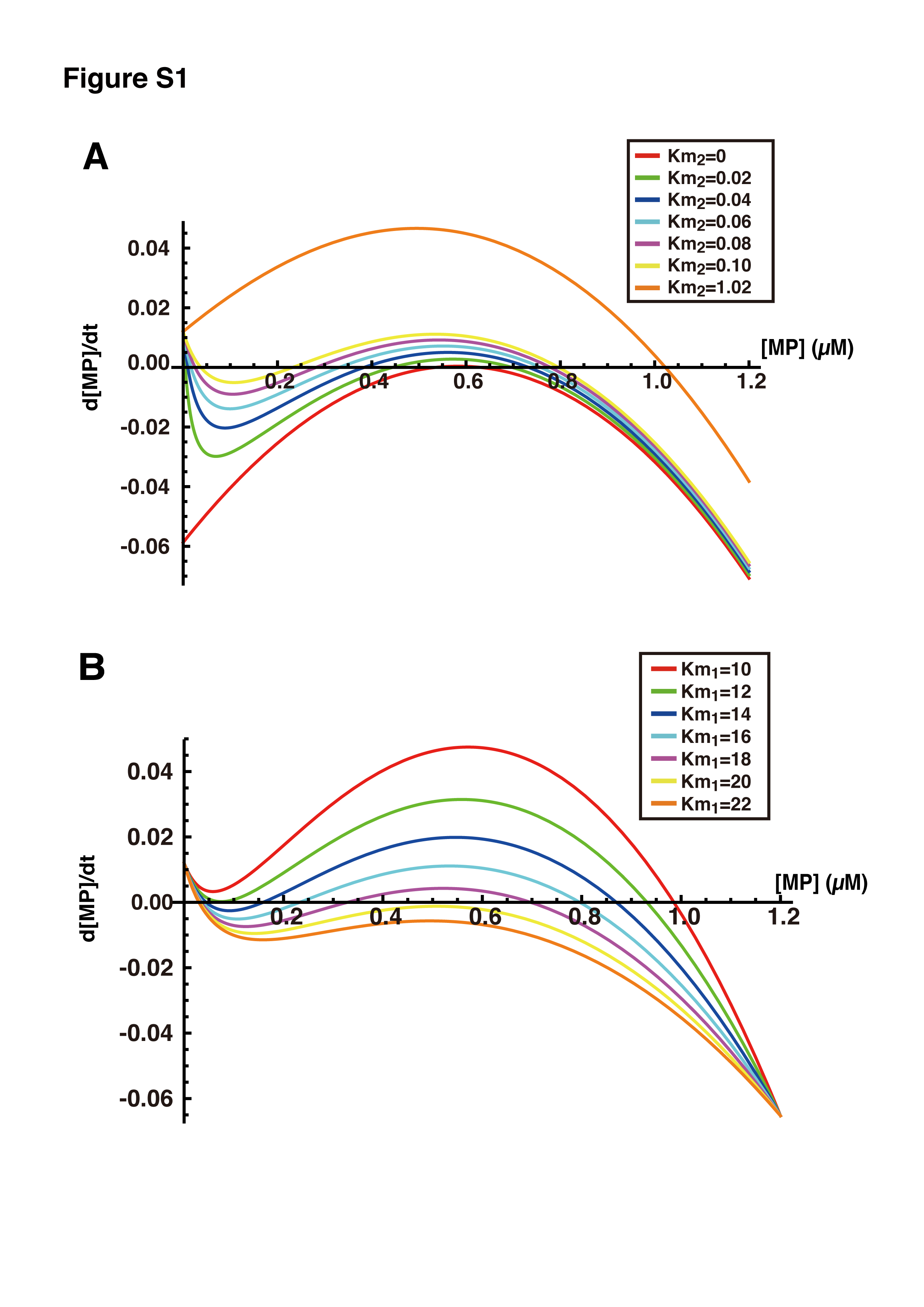

Supplement: Figure S1 — Effects of Rho-kinase and hypothetical pathway. (A) d[MP]/dt was plotted against [MP] using the various Km2 values (Km2 = 0, 0.02, 0.04, 0.06, 0.08, 0.10, and 1.02) for phosphorylation of MYPT1 by Rho-kinase. (B) d[MP]/dt was plotted against [MP] using the various Km1 values (Km1 = 10, 12, 14, 16, 18, 20 and 22) for dephosphorylation of MYPT1 by myosin phosphatase. (TIF) [file pone.0039269.s001.tif]

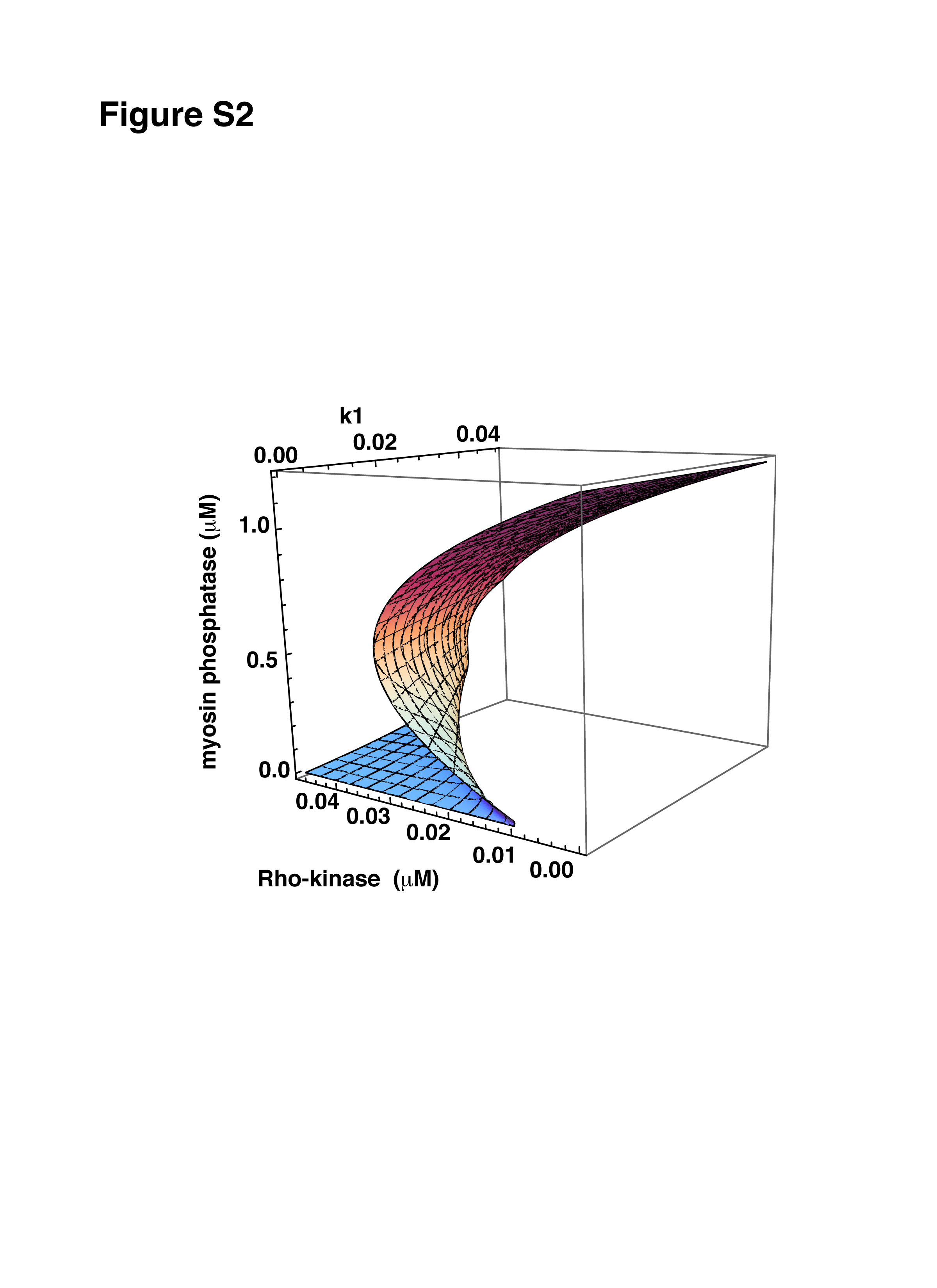

Supplement: Figure S2 — Estimation of Rho-kinase activity and rate constant k1. The steady states of activated myosin phosphatase against Rho-kinase activity and rate constant (k1). X, Y, and Z axes show the Rho-kinase activities, rate constant k1, and the steady states of myosin phosphatase, respectively. (TIF) [file pone.0039269.s002.tif]
